# Efficacy and central mechanism of acupuncture treatment in patients with neck pain: study protocol for a randomized controlled trial

**DOI:** 10.1186/s13063-021-05507-y

**Published:** 2021-08-14

**Authors:** Zhen Gao, Tao Yin, Lei Lan, Dehua Li, Ruirui Sun, Guodong Ha, Caili Jiang, Xin Shao, Zhaoxuan He, Laixi Ji, Fang Zeng

**Affiliations:** 1 Brain Research Center, Acupuncture and Tuina School/The 3rd Teaching Hospital, Chengdu University of Traditional Chinese Medicine, 37# Shierqiao Road, Chengdu, 610075 Sichuan China; 2 Hospital of Chengdu University of Traditional Chinese Medicine, Chengdu, 610072 Sichuan China; 3 Sichuan Integrated Medicine Hospital, Chengdu, 610041 Sichuan China; 4 Graduate Faculty, Shanxi University of Traditional Chinese Medicine, 121# University Street, Jinzhong, 030619 Shanxi China

**Keywords:** Neck pain, Acupuncture, Central mechanism, Functional magnetic resonance imaging, Protocol

## Abstract

**Background:**

Acupuncture is effective for reducing the symptoms of neck pain (NP). However, the underlying mechanisms are not fully elucidated. Based on evaluating the efficacy of two acupuncture prescriptions for treating NP, this study aims to investigate the potential central mechanism of acupuncture treatment for NP by functional magnetic resonance imaging (fMRI).

**Methods:**

This is a randomized controlled trial; 86 patients will be randomly assigned into two acupuncture treatment groups at a ratio of 1:1. The whole study period includes 2 weeks baseline, 2 weeks treatments, and 12 weeks follow-up (4 and 12 weeks after treatment). The pain severity, the neck disability index, the cervical range of motion, and the pressure pain threshold, etc., will be used to evaluate the clinical efficacy of two acupuncture prescriptions for NP treatment. The MRI scans will be performed to detect cerebral activity changes of 20 patients in each group. The clinical data and MRI data will be analyzed, respectively. *Pearson* correlation coefficient will be used to evaluate the association between changes of cerebral activity features and improvement of clinical symptoms.

**Discussion:**

The results will provide further evidence for the clinical application of acupuncture in the treatment of NP.

**Trial registration:**

Chinese Clinical Trial Registry ChiCTR2000040930. Registered on 16 December 2020.

**Supplementary Information:**

The online version contains supplementary material available at 10.1186/s13063-021-05507-y.

## Background

Neck pain (NP), as a common musculoskeletal disorder, has become an important healthcare and social problem due to its high prevalence and heavy economic burden [1]. It is estimated that more than 30% of the population suffer from NP each year [2], and its lifetime prevalence in adults ranges from 14.2 to 71% [3]. Due to the increasing prevalence of NP, the health care costs of NP have rapidly increased [4–6].

In the United States, the cumulative annual treatment costs for lower back pain and NP is about $ 87.6 billion [7]. The treatment of NP has been challenging, because its etiology is complex and the symptoms are easy to recur. Although medications (e.g., muscle relaxants and non-steroidal anti-inflammatory drugs) can effectively alleviate acute pain, their side effects, including hepatotoxicity, etc., cannot be ignored [8]. Therefore, more patients tend to purchase non-drug therapies to relieve symptoms.

Acupuncture has been used to relieve pain for thousands of years in China. As early as 1997, the National Institutes of Health (NIH) recommended acupuncture treatment for pain disorders. Evidence from recent large-scale clinical studies has confirmed that acupuncture can effectively improve NP symptoms [9–14]. However, the underlying mechanisms of acupuncture for NP have not been fully elucidated.

Advances in neuroimaging techniques have enhanced studies into the central mechanisms of acupuncture as an analgesic in vivo. In the last two decades, many neuroimaging studies have been performed to elucidate the mechanisms of acupuncture analgesia, and achieved gratifying results [15–18]. For example, an fMRI study on migraines found that acupuncture was effective in improving abnormally reduced functional activity in the rostral ventromedial medulla/trigeminocervical complex area of patients [19]. Recently, significant alterations in the brain structure and function of NP patients have been reported by neuroimaging studies. Compared to healthy subjects, NP patients shown decreased functional activity in the left sensorimotor cortex and right temporoparietal junction [20], as well as increased functional connectivity between the right dorsolateral prefrontal cortex and the right anterior insula, which are associated with pain intensity [21]. Based on these findings, neuroimaging techniques appear to be useful tools for investigating the central mechanisms of acupuncture therapy for NP. Therefore, by MRI, this study aims to (1) evaluate the therapeutic effects of different acupoints prescription on NP, (2) investigate the changes in cerebral activity elicited by acupuncture treatment, and (3) analyze the possible correlations between the improvement of clinical symptoms and changes in brain activity, so as to explore the potential central mechanism of acupuncture for NP.

## Methods and design

### Study design

This is a randomized controlled trial. This protocol is reported in accordance with the Standard Protocol Items: Recommendations for Intervention Trials (SPIRIT) guidelines (supplementary file 1) and follows the principles of the Consolidated Standards of Reporting Trials (CONSORT) and the Standards for Reporting Interventions in Clinical Trials of Acupuncture (STRICTA) [22, 23]. The protocol has been approved by the Institutional Review Board of the Hospital of Chengdu University of Traditional Chinese Medicine (approved number: 2020KL-007) and registered at Chinese Clinical Trial Registry (registration number: ChiCTR2000040930, protocol version number: V1.0).

**Fig. 1:**
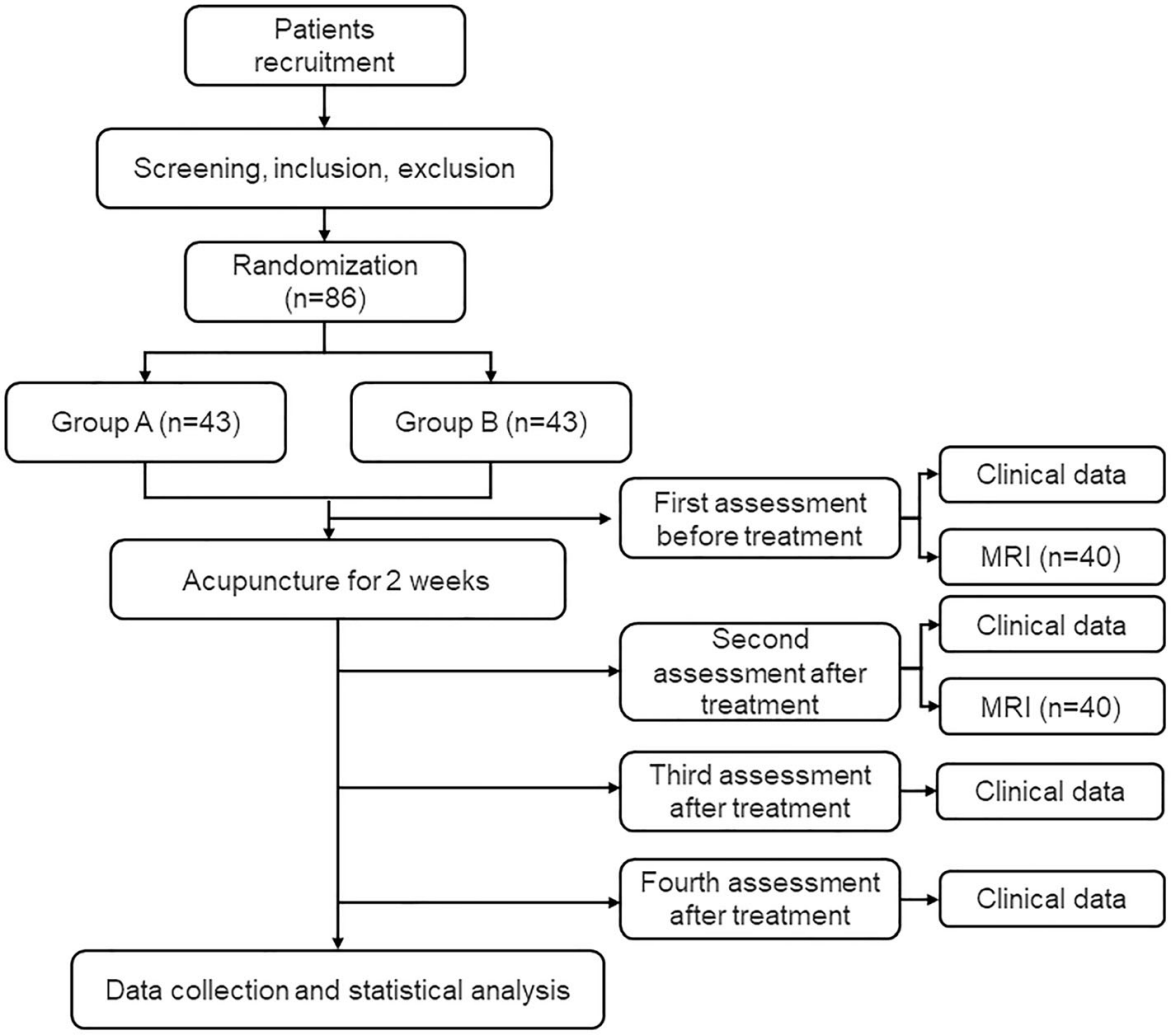
Flowchart of the procedure through the trial. The randomized controlled trial will enroll 86 eligible patients and divide them randomly into two groups at a 1:1 ratio. Twenty patients from each group will be randomly selected for MRI data collection. During the 2-week treatment, the patient will receive 6 acupuncture treatments. Clinical assessment will be performed at 4 time points: baseline, end of treatment, and 6 and 14 weeks after completion. The MRI scans will be performed at baseline and after treatment. Abbreviations: MRI magnetic resonance imaging

This study period last for 16 weeks, which included a 2-week baseline period, a 2-week intervention period, and a 12-week following period. A total of 86 patients with NP will be randomly assigned to the two acupuncture groups with a 1:1 ratio. Twenty patients in each group will be randomly selected to undertake MRI scans. Clinical outcome evaluation and MRI scans will be conducted at baseline and after treatment. The study procedure is outlined in Fig. 1 and Table 1.

**Table 1:** Study schedule for data collection

Period	Baseline		Treatment						Follow-up	
Assessment		1						2	3	4
Time (week)	0	2	3			4			8	16
**Pick-up information**										
Inclusion/exclusion criteria	√									
Informed consent	√									
Vital signs	√									
Medical history	√									
Physical examination	√							√		
**Acupuncture intervention**										
Group A (*n*=43)			√	√	√	√	√	√		
Group B (*n*=43)			√	√	√	√	√	√		
**Clinical assessment**										
VAS	√	√						√	√	√
NDI		√						√	√	√
ROM	√	√						√	√	√
PPT		√						√	√	√
SF-36		√						√	√	√
MSUS		√						√		
SAS and SDS		√						√	√	√
MRI (*n*=40)		√						√		
Laboratory test		√						√		
Adverse event								√		

This is a randomized controlled trial of neuroimaging including a 2-week baseline period, a 2-week treatment period, and a 12-week follow-up period. At baseline, patients will be screened based on inclusion and exclusion criteria; eligible individuals will then sign informed consent and undergo a physical examination. After being randomized into 2 groups, 6 acupuncture treatments will be performed for 2 weeks. Clinical data will be evaluated at baseline, after treatment ends, and at 4 and 12 weeks after completion; MRI scans will be performed at baseline and after treatment. After the treatment, laboratory tests will be performed, including routine blood test, C-reactive protein, and erythrocyte sedimentation tests. Adverse events will be recorded in the CRF at any time during treatment. Abbreviations:MRI magnetic resonance imaging, VAS visual analogue scale, NDI neck disability index, ROM range of motion, PPT pressure pain thresholds, MSUS musculoskeletal ultrasound, SF-36 Short-Form 36-Item Health Survey, SAS Self-rating Anxiety Scale, SDS Self-rating Depression Scale, CRF case report form

### Participants

#### Recruitment strategy

Patients will be recruited at the outpatient of the Hospital of Chengdu University of Traditional Chinese Medicine (TCM) and Sichuan Integrated Medicine Hospital. In addition, patients will also be recruited from the community and campus of Chengdu University of TCM through advertising (posting notices, distributing leaflets, web publishing). Patients who agree to participate in this study will be diagnosed by an orthopedic specialist. The eligible patients will be screened by the research assistant based on the inclusion and exclusion criteria.

#### Inclusion criteria

Participants who match the inclusion criteria will be included: (1) patients with NP as the main complaint and a visual analog scale (VAS) score of pain severity exceeding 4 points but less than 10 points (range 0–10 points), (2) for a duration of ≤ 3 months, (3) aged 18–60 years, and (4) agreeing to sign informed consent.

#### Exclusion criteria

Participants with any of the following conditions will be excluded: (1) they are pregnant or lactating women; (2) they have any organic or metabolic diseases of the digestive, hematopoietic, endocrine, or immune systems, or other severe primary diseases; (3) they have fibromyalgia and other central sensitization pain syndromes; (4) they have local skin damage or severe skin diseases that affect acupuncture manipulation; (5) they combine with mental or neurological diseases; (6) they have received acupuncture for NP within 1 month; (7) they have MRI contraindications, such as heart pacemaker, metallic foreign bodies, or severe claustrophobia, etc.; or (8) they are participating in other clinical trials.

#### Sample size

This study aims to compare the difference of the clinical efficacy of two acupuncture prescriptions (group A and group B). According to the results of the pilot study, the average reduction of VAS score in group A was 2.86±0.99, and the average reduction of VAS score in group B was 2.14±0.99. According to the formula, $n=\frac{2{\sigma}^2}{{\left(\mu 1-\mu 2\right)}^2}\times {\left({\mu}_{\alpha /2}+{\mu}_{\beta}\right)}^2$, with *α* = 0.05 (both sides) and 1 − *β* = 0.80 [24]. The sample size of each group was calculated to be 34, considering a 20% dropout rate; the final sample size was set to 43 per group, making the total of 86 in this study.

There is no widely accepted method for sample size calculation in neuroimaging study. Referring to the previous study [25], 15 participants in each group are the smallest size in neuroimaging studies. Considering a 20% dropout rate and the unavailability of data due to various reasons, this study will randomly select 20 patients from each group for MRI scans.

### Randomization

Randomization will be carried out in two steps: (1) an independent, blinded statistician will generate a random allocation sequence using SPSS 26.0 (IBM, Chicago, IL, USA). The 86 patients will be randomly assigned into group A and group B at a ratio of 1:1; (2) 20 patients in each group will be randomly selected for MRI according to another random sequence. Allocation concealment will be achieved by sequentially numbered, opaque, sealed envelopes.

### Blinding

In this study, the patients, outcome assessors, and statistical analysts will be blinded. The patients will be treated in separate rooms to reduce communication. However, due to the particularity of acupuncture manipulation, blinding operator cannot be achieved.

### Interventions

**Fig. 2:**
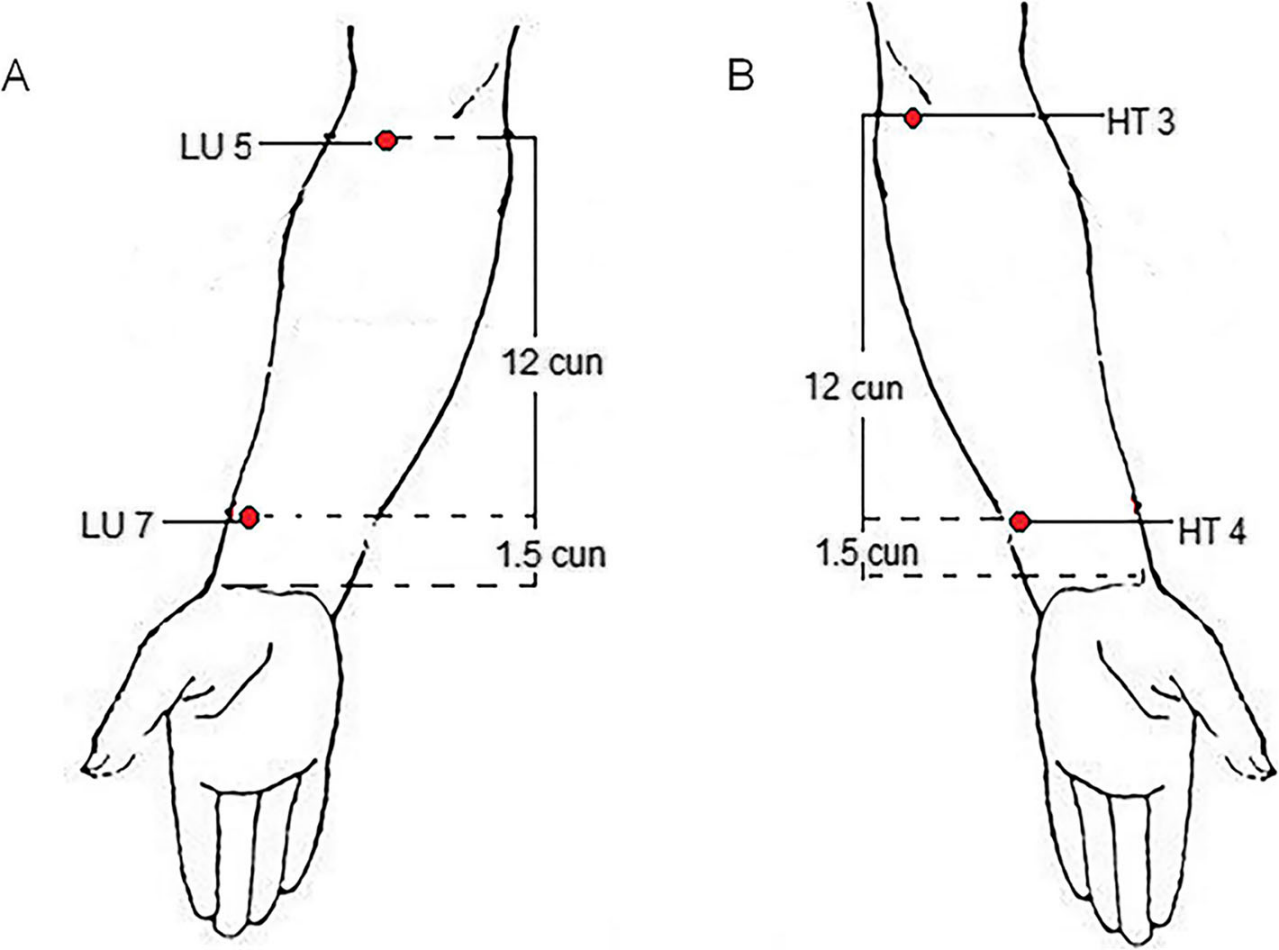
Locations of acupoints. **A** Locations of acupoints (group A). *Lieque* (LU 7), on the radial side of the forearm, above the styloid process of the radius and 1.5 cun above the horizontal stripes of the wrist. *Chize* (LU 5), in the transverse elbow, the radial side of the biceps tendon is sunken. **B** Locations of acupoints (group B). *Lingdao* (HT 4), in the anterior area of the forearm, the radial side of the ulnar carpi flexor tendon is 1.5 cun above the transverse stripes of the carpal palm. *Shaohai* (HT 3), at the midpoint of the line between the medial end of the transverse line of the elbow and the medial epicondyle of the humerus

Patients in group A will receive acupuncture at three acupoints (*Lieque* (LU7), *Chize* (LU5), and neck tenderness point on the affected side) (Fig. 2A). The acupoints of group B (control group) will receive acupuncture at three acupoints (*Shaohai* (HT3), *Lingdao* (HT4), and neck tenderness point on the affected side) (Fig. 2B). Tenderness points will be detected using a PX25 hand-held pressure pain tester (Wagner FPX™ 25).

The acupuncture manipulations are as follows: disposable sterile filiform needles (0.30 × 40 mm, Huatuo Medical Instrument Co., Ltd., China) will be inserted into acupoints at a depth of 20–30 mm after skin disinfection, and the Deqi sensation (a sensation of acid distension or numbness, or other acupuncture sensation) will be achieved [26]. Then, the HANS-200A acupoint nerve stimulator (Nanjing Jisheng Medical Technology Company, Nanjing city, China) will connect LU7 and LU5 of group A or HT3 and HT4 of group B respectively in the two groups for 30 min with dilatational waves (2–100 Hz, 1 mA). Patients will receive a total of 6 sessions of acupuncture in two weeks, with 3 sessions per week.

Acupuncture treatment will be performed by acupuncturists who have held a practitioner license for more than 3 years.

### Concomitant medications

Patients will be instructed not to take any other analgesic medications for NP during this study. In cases of severe NP, ibuprofen (300 mg per capsule with sustained release) will be allowed as rescue medication and should be recorded on the case report form (CRF). Patients are also asked to report to the researchers if they take any concomitant medications during the study, and to record the name, dose, and date of the medications used.

### Outcomes

The measurements will be conducted by independent assessors who have been trained before the study. The primary outcome is the change of VAS scores at baseline and after the end of treatment. VAS scores range from 0 to 10, with 0 indicating no pain and 10 indicating pain is unbearable. The secondary outcome measurements include neck disability index (NDI), Short-Form 36-Item Health Survey (SF-36), Self-rating Anxiety Scale (SAS), Self-rating Depression Scale (SDS), range of motion (ROM), and pressure pain threshold (PPT) will be evaluated at baseline, after treatment, and at 4 and 12 weeks after the end of treatment. In addition, we will perform musculoskeletal ultrasound (MSUS) before and after treatment. Among them, NDI is the most widely used clinical tool for self-assessment of disability caused by NP [27]. The SAS and SDS are the commonly used psychometric tools to measure the severity of anxiety and depression [28, 29]. SF-36 is a general health-based survey of quality of life, which can be self-administered by the patient with reliability [30]. ROM is one of the quantitative outcomes assessing the extent and degree of joint motor impairment. The changes of the tenderness threshold can quantitatively reflect the improvement of the pain severity [31]. Meanwhile, MSUS is an important method for clinical diagnosis of rehabilitation medicine, tracking disease progression and assessing curative effect [32].

### Patient safety

Adverse events during the study period will be reported in the CRF. The record should include the time, reason, clinical symptoms, signs of the adverse event, and the corresponding emergency treatment plan, as well as abnormal laboratory tests with clinical significance. In addition, all events will be evaluated for their relevance to the intervention and severity, according to the clinician.

### MRI data acquisition

The resting-state fMRI (RS-fMRI) will be performed at the baseline and the end of treatment, respectively. Patients will be asked to do not drink tea, coffee, or alcohol, avoid strenuous exercise, and ensure adequate sleep before the scan.

Patients will undergo RS-fMRI scan with a 3.0T MR scanners (Siemens 3T Tim trio, Erlangen, Germany) at the West China Hospital of Sichuan University. Patients will be asked to stay awake and to keep their heads still during the scan, with their eyes closed and ears plugged. Scans will be performed with the following procedures: localizer, three-dimensional T1-weighted imaging (3D-T1WI), blood oxygenation level-dependent fMRI (BOLD-fMRI) and diffusion tensor imaging (DTI) sequence. The 3D-T1WI scanning parameters will be as follows: repetition time (TR) = 6.008 ms, echo time (TE) = 1.7 ms, data matrix = 256 × 256, field of view (FOV) = 256 × 256 mm^2^. The BOLD-fMRI scanning parameters will be as follows: 31 contiguous slices with a slice thickness of 5 mm, TR = 2000 ms, TE = 30 ms, FOV = 240 × 240 mm^2^, Matrix = 64 × 64, flip angle (FA) = 90°, total volumes = 240. The DTI data will be acquired using a single-shot echo planar image sequence with the following parameters: FOV = 256 × 256 mm^2^, TR = 8500 ms, matrix = 128 × 128, slice thickness = 2 mm with no gap. Two diffusion-weighted sequences were acquired using gradient values b = 1000 s/mm^2^ and b = 0 s/mm^2^ with the diffusion-sensitizing gradients applied in 64 non-collinear directions.

### Data management

An electronic entry database will be established based on the CRFs project, and the outcome assessors will fill in relevant information in a timely and accurate manner according to the requirements of CRFs. Only outcome assessors have access to the CRFs and perform double-data entry. The Evidence-based Medicine Center of the Hospital of Chengdu University of TCM will be responsible for monitoring the study and data every 3 months.

### Data analysis

#### Clinical data analysis

The comprehensive effectiveness analysis will use the per protocol set (PPS) and the full analysis set (FAS). The FAS population will be used as the primary population for all efficacy analyses. The safety set (SS) included all subjects who received at least one treatment after randomization. When the results of PPS analysis and FAS analysis are inconsistent, PPS and FAS are discussed separately to find out the reasons for the inconsistency. Missing data will be handled using the last-observation-carried-forward (LOCF) method.

Statistical analysis will be conducted using SPSS 26.0 (IBM, Chicago, IL, USA) statistical software. *P* < 0.05 (two-sided) is considered statistically significant. Quantitative data are described with mean ± standard deviation (SD). Qualitative data are described with frequency and percentage (*N*, %). Student’s *t-*test and chi-square test will be used to compare the differences between groups at baseline. The assessment of the difference between the two groups will use repeated measures analysis of covariance (ANCOVA), taking into account the baseline values of age, sex and disease duration as covariates. Clinical data on skewed distribution will be compared using a non-parametric test.

#### BOLD-fMRI data analysis

The fMRI data preprocessing and comparison will be performed using SPM12 software (http://www.fil.ion.ucl.ac.uk/spm/) based on MATLAB 2018a (Mathworks, Inc., USA). The steps including: format conversion, slice timing correction, head motion correction, spatial standardization, linear trend removal, spatial smoothing, and band-pass filtering. After pre-processing, the amplitude of low frequency fluctuation and regional homogeneity will be calculated to reflect the local cerebral functional activity, and the seeds-based functional connection analysis will be performed to reflect the functional integration of the brain. ANOVA will be used for comparisons between the two groups, with multiple comparison corrections.

Furthermore, the *Pearson* correlation analysis will be conducted to assess the associations between the changes of fMRI and the improvement of clinical symptoms.

## Discussion

To the best of our knowledge, this is the first randomized controlled study to investigate the clinical efficacy and underlying mechanisms of different acupuncture prescriptions for NP. The results of this study are beneficial for the prescription selection of acupuncture for treating NP and elucidate on the mechanisms of acupuncture for NP.

### Selection of acupoint prescription

Many clinical studies have been performed on acupuncture for NP, and its efficacy has been identified. In clinical practice, the combination of local and distal points is a basic principle of acupoint prescriptions, but the number of prescriptions and acupoints involved is enormous. Therefore, in this study, two different prescriptions will be selected to compare their efficacy and possible mechanisms. Neck tenderness point will be selected as the basic acupoint, which is the most commonly used local acupoint in NP treatment. LU7 and LU5, located in the forearm, will be selected as the distal points in group A. These two points are the effective and common acupoints for neck diseases based on the traditional Chinese medicine theories and clinical practice [33–35]. HT4 and HT3, also located in the forearm, will be selected as the distal points in group B. These two acupoints are located on the same nerve segment as those in group A. This is a commonly available method for determining a control group in acupuncture studies and has been proven to be effective in blinding patients in many studies [36, 37].

### The combination of subjective and objective outcome measures

Pain is an unpleasant sensory and emotional perception that is associated with actual or underlying tissue pathology [38]. Human behavior, psychological, and social factors play a crucial role in the long-term maintenance of pain [39]. Therefore, the multidimensional nature of pain requires that the measurement of NP should include both subjective outcomes and objective outcomes. In this study, subjective outcomes including VAS, NDI, SAS, and SDS will be used to assess pain perception, dysfunction, and emotional state of the patients. Objective outcomes such as tenderness threshold, musculoskeletal ultrasound, and ROM will be used to evaluate the symptom improvement in the NP patients. The combination of subjective and objective outcomes can be more complementary to assess the efficacy from multiple perspectives and reduce the occurrence of potential bias, so as to improve the quality of clinical evidence.

### Application of neuroimaging technology

Previous neuroimaging studies have demonstrated that patients with NP exhibit abnormalities in brain activity and structure. For example, Chen et al. [40] found increased spontaneous brain activity in the supplementary motor area of NP patients and subsequently suggested a correlation between abnormality of the salience network with the disease. Another single fMRI-based case control study associated NP with activation and/or recall of pain memory [41]. Elsewhere, a voxel-based morphometry study performed on NP patients revealed the loss of bilateral clusters of gray matter in the sensorimotor cortex and pulvinar nucleus with NP pathophysiology [42].

Moreover, it has been reported that central integration is the key determinant of the effectiveness of acupuncture [43]. With previous studies, performed on individuals with shoulder pain [44], fibromyalgia [45], and low back pain [46], as well as knee osteoarthritis [15] indicating that regulation of brain activity might be an important factor influencing acupuncture’s modulating effects on pain. For example, acupuncture has been shown to effectively reduce pain by modulating brain regions related to both sensory-discriminative and emotional aspects [47]. Such regions were found to be important for the therapeutic effects of acupuncture on NP [40]. Based on these evidences, we hypothesize that acupuncture can effectively treat NP by modulating alteration of brain activity.

This trial is the first study to use fMRI to explore the central mechanisms of different acupuncture prescriptions in the treatment of NP. The results of this trial will help to provide visualization evidence for the clinical application of acupuncture for NP treatment.

### Trial status

The trial is currently in the recruitment phase and the study is expected to end in December 2021.

## Supplementary Information

**Additional file 1.** SPIRIT 2013 Checklist: Recommended items to address in a clinical trial protocol and related documents.

## Data Availability

Not applicable.

## Abbreviations

3D-T1WI: Three-dimensional T1-weighted imaging
ANCOVA: Analysis of covariance
BOLD-fMRI: Blood oxygenation level-dependent fMRI
RS-fMRI: Resting state functional magnetic resonance imaging
CRF: Case report form
DTI: Diffusion tensor imaging
FA: Flip angle
FAS: Full analysis set
FDR: False discovery rate
FOV: Field of view
GLM: General linear model
HbO: Oxygenated hemoglobin
HbR: Deoxygenated hemoglobin
LOCF: Last observation carry-forward
MRI: Magnetic resonance imaging
MSUS: Musculoskeletal ultrasound
NDI: Neck disability index
NP: Neck pain
NIH: National Institutes of Health
PPS: Per protocol set
PPT: Pressure pain threshold
ROM: Range of motion
SD: Standard deviation
SF-36: Short-form 36-item Health Survey
SAS: Self-Rating Anxiety Scale
SDS: Self-Rating Depression
SS: Safety set
TCM: Traditional Chinese medicine
TE: Echo time
TR: Repetition time
VAS: Visual analogue scale

## References

[1] Safiri S, Kolahi AA, Hoy D, Buchbinder R, Mansournia MA, Bettampadi D, et al. Global, regional, and national burden of neck pain in the general population, 1990-2017: systematic analysis of the Global Burden of Disease Study 2017. BMJ (Clinical research ed). 2020;368:m791. 10.1136/bmj.m791 PMC7249252 32217608

[2] Cohen SP (2015). Epidemiology, diagnosis, and treatment of neck pain. Mayo Clinic proceedings..

[3] Fejer R, Kyvik KO, Hartvigsen J (2006). The prevalence of neck pain in the world population: a systematic critical review of the literature. European spine journal : official publication of the European Spine Society, the European Spinal Deformity Society, and the European Section of the Cervical Spine Research Society..

[4] Hurwitz EL, Randhawa K, Yu H, Côté P, Haldeman S. The Global Spine Care Initiative: a summary of the global burden of low back and neck pain studies. European spine journal : official publication of the European Spine Society, the European Spinal Deformity Society, and the European Section of the Cervical Spine Research Society. 2018;27(Suppl 6):796-801, DOI: 10.1007/s00586-017-5432-9. 10.1007/s00586-017-5432-9 29480409

[5] Hoy D, March L, Woolf A, Blyth F, Brooks P, Smith E, Vos T, Barendregt J, Blore J, Murray C, Burstein R, Buchbinder R (2014). The global burden of neck pain: estimates from the global burden of disease 2010 study. Annals of the rheumatic diseases..

[6] Basson CA, Olivier B, Rushton A (2019). Neck pain in South Africa: an overview of the prevalence, assessment and management for the contemporary clinician. The South African journal of physiotherapy..

[7] Dieleman JL, Baral R, Birger M, Bui AL, Bulchis A, Chapin A, Hamavid H, Horst C, Johnson EK, Joseph J, Lavado R, Lomsadze L, Reynolds A, Squires E, Campbell M, DeCenso B, Dicker D, Flaxman AD, Gabert R, Highfill T, Naghavi M, Nightingale N, Templin T, Tobias MI, Vos T, Murray CJL (2016). US spending on personal health care and public health, 1996-2013. Jama..

[8] Cohen SP, Hooten WM. Advances in the diagnosis and management of neck pain. BMJ (Clinical research ed). 2017;358:j3221. 10.1136/bmj.j3221 28807894

[9] Irnich D, Behrens N, Molzen H, König A, Gleditsch J, Krauss M, Natalis M, Senn E, Beyer A, Schöps P (2001). Randomised trial of acupuncture compared with conventional massage and "sham" laser acupuncture for treatment of chronic neck pain. BMJ (Clinical research ed)..

[10] MacPherson H, Tilbrook H, Richmond S, Woodman J, Ballard K, Atkin K, Bland M, Eldred J, Essex H, Hewitt C, Hopton A, Keding A, Lansdown H, Parrott S, Torgerson D, Wenham A, Watt I (2015). Alexander technique lessons or acupuncture sessions for persons with chronic neck pain: a randomized trial. Ann Internal Med.

[11] Wong JJ, Shearer HM, Mior S, Jacobs C, Côté P, Randhawa K, Yu H, Southerst D, Varatharajan S, Sutton D, van der Velde G, Carroll LJ, Ameis A, Ammendolia C, Brison R, Nordin M, Stupar M, Taylor-Vaisey A (2016). Are manual therapies, passive physical modalities, or acupuncture effective for the management of patients with whiplash-associated disorders or neck pain and associated disorders? An update of the Bone and Joint Decade Task Force on Neck Pain and Its Associated Disorders by the OPTIMa collaboration. The spine journal : official journal of the North American Spine Society..

[12] Yuan QL, Guo TM, Liu L, Sun F, Zhang YG (2015). Traditional Chinese medicine for neck pain and low back pain: a systematic review and meta-analysis. PloS one..

[13] Chen L, Li M, Fan L, Zhu X, Liu J, Li H, Xu Z, Chen J, Liang Z, Liu Z, Feng L, Chen X, He Q, Chen X, Ou A, He J, Ma R, Ning B, Jiang L, Li S, Fu W (2021). Optimized acupuncture treatment (acupuncture and intradermal needling) for cervical spondylosis-related neck pain: a multicenter randomized controlled trial. Pain..

[14] Ho LF, Lin ZX, Leung AWN, Chen L, Zhang H, Ng BFL, Ziea ETC, Guo Y (2017). Efficacy of abdominal acupuncture for neck pain: a randomized controlled trial. PloS one..

[15] Kong J, Wang Z, Leiser J, Minicucci D, Edwards R, Kirsch I, Wasan AD, Lang C, Gerber J, Yu S, Napadow V, Kaptchuk TJ, Gollub RL (2018). Enhancing treatment of osteoarthritis knee pain by boosting expectancy: a functional neuroimaging study. NeuroImage Clinical..

[16] Cao J, Tu Y, Orr SP, Lang C, Park J, Vangel M, et al. Analgesic effects evoked by real and imagined acupuncture: a neuroimaging study. Cerebral cortex (New York, NY : 1991). 2019;29(8):3220-31. 10.1093/cercor/bhy190 PMC7302519 30137262

[17] Kong J, Kaptchuk TJ, Polich G, Kirsch I, Vangel M, Zyloney C, Rosen B, Gollub R (2009). Expectancy and treatment interactions: a dissociation between acupuncture analgesia and expectancy evoked placebo analgesia. NeuroImage..

[18] Kong J, Kaptchuk TJ, Polich G, Kirsch I, Vangel M, Zyloney C, Rosen B, Gollub RL (2009). An fMRI study on the interaction and dissociation between expectation of pain relief and acupuncture treatment. NeuroImage..

[19] Li Z, Zeng F, Yin T, Lan L, Makris N, Jorgenson K, Guo T, Wu F, Gao Y, Dong M, Liu M, Yang J, Li Y, Gong Q, Liang F, Kong J (2017). Acupuncture modulates the abnormal brainstem activity in migraine without aura patients. NeuroImage Clinical.

[20] Chen J, Wang Z, Tu Y, Liu X, Jorgenson K, Ye G, Lin C, Liu J, Park J, Lang C, Liu B, Kong J (2018). Regional homogeneity and multivariate pattern analysis of cervical spondylosis neck pain and the modulation effect of treatment. Front Neuroscience.

[21] Ihara N, Wakaizumi K, Nishimura D, Kato J, Yamada T, Suzuki T, Hashiguchi S, Terasawa Y, Kosugi S, Morisaki H (2019). Aberrant resting-state functional connectivity of the dorsolateral prefrontal cortex to the anterior insula and its association with fear avoidance belief in chronic neck pain patients. PloS one.

[22] Schulz KF, Altman DG, Moher D (2011). CONSORT 2010 statement: updated guidelines for reporting parallel group randomised trials. Int J Surgery (London, England).

[23] MacPherson H, Altman DG, Hammerschlag R, Youping L, Taixiang W, White A, Moher D, on behalf of the STRICTA Revision Group (2010). Revised STandards for Reporting Interventions in Clinical Trials of Acupuncture (STRICTA): extending the CONSORT statement. PLoS medicine..

[24] Chow S, Shao J, Wang H (2008). Sample size calculations in clinical research.

[25] Hayasaka S, Peiffer AM, Hugenschmidt CE, Laurienti PJ (2007). Power and sample size calculation for neuroimaging studies by non-central random field theory. NeuroImage..

[26] Yuan HW, Ma LX, Qi DD, Zhang P, Li CH, Zhu J (2013). The historical development of deqi concept from classics of traditional chinese medicine to modern research: exploitation of the connotation of deqi in chinese medicine. Evidence-based complementary and alternative medicine : eCAM..

[27] Blanpied PR, Gross AR, Elliott JM, Devaney LL, Clewley D, Walton DM, Sparks C, Robertson EK (2017). Neck Pain: Revision 2017. J Orthopaedic Sports Physical Therapy.

[28] Zung WW (1971). A rating instrument for anxiety disorders. Psychosomatics..

[29] Zung WW (1965). A SELF-RATING DEPRESSION SCALE. Archives of general psychiatry..

[30] Patel AA, Donegan D, Albert T. The 36-item short form. The Journal of the American Academy of Orthopaedic Surgeons. 2007;15(2):126-134, DOI: 10.5435/00124635-200702000-00007. 10.5435/00124635-200702000-00007 17277259

[31] Walton DM, Macdermid JC, Nielson W, Teasell RW, Nailer T, Maheu P (2011). A descriptive study of pressure pain threshold at 2 standardized sites in people with acute or subacute neck pain. J Orthopaedic Sports Physical Therapy.

[32] Klauser AS, Miyamoto H, Bellmann-Weiler R, Feuchtner GM, Wick MC, Jaschke WR (2014). Sonoelastography: musculoskeletal applications. Radiology..

[33] Wang YJ, Zhang LJ, Song K. [Verification of the theory of "Lieque (LU 7) for the disorders of the head and neck" based on infrared thermography]. Zhongguo zhen jiu = Chinese acupuncture & moxibustion. 2019;39(2):169-72. 10.13703/j.0255-2930.2019.02.016 30942036

[34] Yao XJ, Liu JW. [Observation on clinical efficacy of acute pain treated with the intervention of different time of needle retention]. Zhongguo zhen jiu = Chinese acupuncture & moxibustion. 2013;33(11):985-8. 24494283

[35] Lei Q (2020). Zheng's Wentong acupuncture method treats 35 cases of nerve root cervical spondylopathy. Traditional Chinese Medicinal Research..

[36] Zheng H, Tian XP, Li Y, Liang FR, Yu SG, Liu XG, Tang Y, Yang XG, Yan J, Sun GJ, Chang XR, Zhang HX, Ma TT, Yu SY (2009). Acupuncture as a treatment for functional dyspepsia: design and methods of a randomized controlled trial. Trials..

[37] Zhao L, Li D, Zheng H, Chang X, Cui J, Wang R, Shi J, Fan H, Li Y, Sun X, Zhang F, Wu X, Liang F (2019). Acupuncture as adjunctive therapy for chronic stable angina: a randomized clinical trial. JAMA internal medicine..

[38] Wang VC, Mullally WJ (2020). Pain neurology. The American journal of medicine..

[39] Montoya P, Larbig W, Braun C, Preissl H, Birbaumer N (2004). Influence of social support and emotional context on pain processing and magnetic brain responses in fibromyalgia. Arthritis and rheumatism..

[40] Chen W, Hou X, Chen J, Zhang D, Ye G, Lin C, et al. [MRI pain matrix regional homogeneity in cervical spondylosis of neck type treated with acupuncture at multiple acupoints]. Zhongguo zhen jiu = Chinese acupuncture & moxibustion. 2015;35(10):1005-9. 26790206

[41] Beinert K, Mouthon A, Keller M, Mouthon M, Annoni JM, Taube W (2017). Neural correlates of maladaptive pain behavior in chronic neck pain - a single case control fMRI study. Pain physician..

[42] Bernabéu-Sanz Á, Mollá-Torró JV, López-Celada S, Moreno López P, Fernández-Jover E (2020). MRI evidence of brain atrophy, white matter damage, and functional adaptive changes in patients with cervical spondylosis and prolonged spinal cord compression. European radiology.

[43] Zhang Y, Zhang H, Nierhaus T, Pach D, Witt CM, Yi M (2019). Default mode network as a neural substrate of acupuncture: evidence, challenges and strategy. Frontiers in neuroscience..

[44] Yan CQ, Huo JW, Wang X, Zhou P, Zhang YN, Li JL (2020). Different degree centrality changes in the brain after acupuncture on contralateral or ipsilateral acupoint in patients with chronic shoulder pain: a resting-state fMRI study. Neural plasticity..

[45] Mawla I, Ichesco E, Zöllner HJ, Edden RAE, Chenevert T, Buchtel H, et al. Greater somatosensory afference with acupuncture increases primary somatosensory connectivity and alleviates fibromyalgia pain via insular GABA: a randomized neuroimaging trial. Arthritis & rheumatology (Hoboken, NJ). 2020. 10.1002/art.41620 PMC8197768 33314799

[46] Makary MM, Lee J, Lee E, Eun S, Kim J, Jahng GH, Kim K, Youn YS, Lee JH, Park K (2018). Phantom acupuncture induces placebo credibility and vicarious sensations: a parallel fMRI study of low back pain patients. Scientific reports.

[47] Yu SW (2019). Lin SH, Tsai CC, Chaudhuri KR, Huang YC, Chen YS, et al. Acupuncture effect and mechanism for treating pain in patients with Parkinson’s disease. Frontiers in neurology..

